# Multifunctional surface of the nano-morphic PEEK implant with enhanced angiogenic, osteogenic and antibacterial properties

**DOI:** 10.1093/rb/rbae067

**Published:** 2024-06-17

**Authors:** Jiajia Zhang, Tongtong Ma, Xueye Liu, Xiaoran Zhang, Wenqing Meng, Junling Wu

**Affiliations:** Department of Prosthodontics, School and Hospital of Stomatology, Cheeloo College of Medicine, Shandong University & Shandong Key Laboratory of Oral Tissue Regeneration & Shandong Engineering Research Center of Dental Materials and Oral Tissue Regeneration & Shandong Provincial Clinical Research Center for Oral Diseases, No.44-1 Wenhua Road West, 250012, Jinan, Shandong, China; Department of Prosthodontics, School and Hospital of Stomatology, Cheeloo College of Medicine, Shandong University & Shandong Key Laboratory of Oral Tissue Regeneration & Shandong Engineering Research Center of Dental Materials and Oral Tissue Regeneration & Shandong Provincial Clinical Research Center for Oral Diseases, No.44-1 Wenhua Road West, 250012, Jinan, Shandong, China; Department of Prosthodontics, School and Hospital of Stomatology, Cheeloo College of Medicine, Shandong University & Shandong Key Laboratory of Oral Tissue Regeneration & Shandong Engineering Research Center of Dental Materials and Oral Tissue Regeneration & Shandong Provincial Clinical Research Center for Oral Diseases, No.44-1 Wenhua Road West, 250012, Jinan, Shandong, China; Department of Prosthodontics, School and Hospital of Stomatology, Cheeloo College of Medicine, Shandong University & Shandong Key Laboratory of Oral Tissue Regeneration & Shandong Engineering Research Center of Dental Materials and Oral Tissue Regeneration & Shandong Provincial Clinical Research Center for Oral Diseases, No.44-1 Wenhua Road West, 250012, Jinan, Shandong, China; Department of Prosthodontics, School and Hospital of Stomatology, Cheeloo College of Medicine, Shandong University & Shandong Key Laboratory of Oral Tissue Regeneration & Shandong Engineering Research Center of Dental Materials and Oral Tissue Regeneration & Shandong Provincial Clinical Research Center for Oral Diseases, No.44-1 Wenhua Road West, 250012, Jinan, Shandong, China; Department of Prosthodontics, School and Hospital of Stomatology, Cheeloo College of Medicine, Shandong University & Shandong Key Laboratory of Oral Tissue Regeneration & Shandong Engineering Research Center of Dental Materials and Oral Tissue Regeneration & Shandong Provincial Clinical Research Center for Oral Diseases, No.44-1 Wenhua Road West, 250012, Jinan, Shandong, China

**Keywords:** polyetheretherketone, implant, Co ions, PTH (1-34), biomedical coatings

## Abstract

Polyetheretherketone (PEEK) is a high-performance polymer suitable for use in biomedical coatings. The implants based on PEEK have been extensively studied in dental and orthopedic fields. However, their inherent inert surfaces and poor osteogenic properties limit their broader clinical applications. Thus, there is a pressing need to produce a multifunctional PEEK implant to address this issue. In response, we developed sulfonated PEEK (sPEEK)-Cobalt-parathyroid hormone (PTH) materials featuring multifunctional nanostructures. This involved loading cobalt (Co) ions and PTH (1-34) protein onto the PEEK implant to tackle this challenge. The findings revealed that the controlled release of Co^2+^ notably enhanced the vascular formation and the expression of angiogenic-related genes, and offered antimicrobial capabilities for sPEEK-Co-PTH materials. Additionally, the sPEEK-Co-PTH group exhibited improved cell compatibility and bone regeneration capacity in terms of cell activity, alkaline phosphatase (ALP) staining, matrix mineralization and osteogenic gene expression. It surpassed solely sulfonated and other functionalized sPEEK groups, demonstrating comparable efficacy even when compared to the titanium (Ti) group. Crucially, animal experiments also corroborated the significant enhancement of osteogenesis due to the dual loading of cobalt ions and PTH (1-34). This study demonstrated the potential of bioactive Co^2+^ and PTH (1-34) for bone replacement, optimizing the bone integration of PEEK implants in clinical applications.

## Graphical abstract

**Figure:**
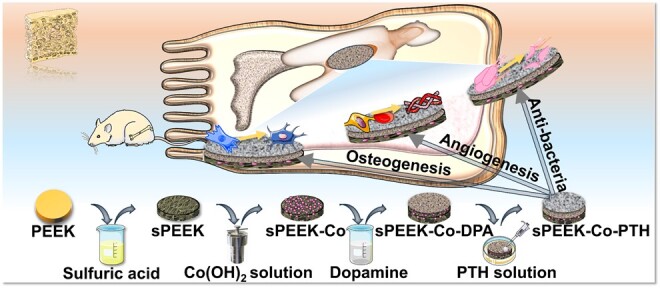

## Introduction

As a promising alternative hard tissue material in dental and orthopedic fields, polyetheretherketone (PEEK) has recently drawn more attention. PEEK, a high-temperature thermoplastic polymer, possesses superior properties, including remarkable chemical stability, outstanding biocompatibility, mechanical characteristics and intrinsic radiolucency [1, 2]. Moreover, compared with titanium (Ti) and its alloys as mainstream bone implant materials, PEEK exhibits a much lower elastic modulus (3–4 GPa) akin to that of human bone [3, 4]. This alleviates the stress shielding effect caused by mechanical mismatch, thus reducing the bone resorption of the peri-implant caused by disuse [5]. However, regrettably, its biological inertness leads to fibrous encapsulation surrounding the PEEK implants instead of effective osseointegration [6, 7]. On the other hand, potential bacterial contamination of PEEK after implantation *in vivo* hampers the clinical application of PEEK [6, 8]. Thus, surface modification of PEEK is desirable to surmount these restrictions without diminishing its inherent advantages.

The success criterion of the implant relies on the effective bone integration between the PEEK implant and the bone tissue. Over the recent decades, considerable attempts have been undertaken to enhance the biocompatibility of PEEK, such as changing the surface micro-nano structure and grafting active functional groups and bioactive agents onto the PEEK surface. It has been widely proven that constructing a 3D porous surface on PEEK could enhance its biological activity. Based on this, some bioactive materials can be used to improve the bone integration capacity of the porous PEEK, such as bone morphogenetic protein 2 [9], osteogenic growth peptide [10], albumin [11], etc. As an active sequence associated with bone remodeling, human parathyroid hormone (PTH) (1-34) has received approval from the US Food and Drug Administration for clinical use in treating bone defects and osteoporosis [12]. PTH (1-34) maintains calcium and phosphorus homeostasis and promotes the proliferation and differentiation of osteoblast cells, thus increasing bone mineral density, trabecular number and bone connectivity [13]. Growing researchers have recently indicated that local PTH (1-34) application benefitted bone regeneration [14, 15], providing theoretical support for combining it with implants to promote osseointegration. Inspired by the mussel, self-polymerized polydopamine can be utilized to functionalize the surfaces of PEEK [16, 17]. Polydopamine coatings can exhibit outstanding biocompatibility, and serve as a ‘bridge’ to bond diverse bioactive molecules for broad applications [16, 18]. Therefore, we employed a polydopamine-aided deposition approach to prepare PEEK coated with PTH (1-34), aiming to investigate its favorable osteogenic properties in this study. In addition to osteogenic activity, angiogenesis capacity also directly influences bone integration and prolonged functionalization of PEEK implants [19]. This is because a vascularized network enables the delivery of osteoblast-related cells and factors and provides nutrients and minerals to promote the survival and mineralization of osteoblasts [20, 21]. Besides, the evidence demonstrates that hastened angiogenesis is propitious to earlier osseointegration [22]. The current studies have focused on combining trace amounts of therapeutic metal cations, specifically cobalt ions (Co^2+^), with some biomaterials, such as bioactive glasses [23], hydrogels [24], hydroxyapatites [25] and bioceramics [26], to improve their angiogenesis induction potential. By simulating an anoxic environment, Co^2+^ can stabilize hypoxia-inducible factor 1α (HIF-1α) and upregulate the expression of vascular endothelial growth factor (VEGF) in cells. Consequently, this leads to the signaling cascade associated with angiogenesis, which can contribute to bone integration of implants [22, 27]. Interestingly, Jiang *et al.* [28] found that while promoting osteogenesis, PTH (1-34) treatment could also stimulate the secretion of VEGF and transforming growth factor-β1(TGF-β1) and recruit vascular endothelial cells. Considering that perspective, combining Co^2+^ and PTH (1-34) may synergistically promote angiogenesis and osteogenesis.

Implant-associated infection is another challenge to realizing optimal osseointegration of PEEK implants, resulting from microbial adhesion and biofilm generation [29]. Bacterial invasion inhibits the capacity of osteogenic-related cells and factors, including alkaline phosphatase (ALP), runt-related transcription factor 2 (Runx2) and osterix [30], ultimately leading to implant failure. Therefore, it is critical to confer antimicrobial abilities to PEEK implants. Fortunately, the Co^2+^ ions can enhance angiogenesis capacity and inhibit bacteria, such as *Escherichia coli* (*E. coli*) [31], *Pseudomonas aeruginosa* [32], and *Staphylococcus aureus* (*S. aureus*) [31–33], by lipid peroxidation and breaking down their cell walls. Although PTH (1-34) or Co^2+^ has been extensively used in biomaterials, no comprehensive work has been published on angiogenesis, osteogenesis and antimicrobial properties of PEEK modified by Co^2+^ and PTH (1-34). Furthermore, multifaceted strategies for enhancing the osteogenic, angiogenic properties and antimicrobial capabilities of PEEK remain limited in current researches.

In this study, we aimed to address the challenge of insufficient osseointegration induced by the surface inertness of PEEK implants and overcome the bacterial contamination risks. Based on the aforementioned considerations, we prepared the PTH (1-34) and Co^2+^ co-functionalized porous PEEK implants for the first time, which possessed triple bioactivities, i.e. angiogenesis, osteogenesis and antimicrobial properties, thus benefiting efficient osseointegration. Both *in vitro* and *in vivo* assessments were conducted to substantiate the biological efficacy of our suggested design program.

## Materials and methods

### Preparation of materials

Biomedical grade PEEK plates were supplied by Jiangsu Junhua Co., Ltd, China, and pure Ti discs (TA2) were supplied by Baoji Taiyuxin Metal Material Co., Ltd, China. Additionally, the PEEK and Ti plates with a size of Φ9 × 2 mm^3^ and Φ20 × 2 mm^3^ were used for the material characterization experiments and *in vitro* tests (Figure S1). These rod-shaped materials with a size of Φ2 × 7 mm^3^ were used for the *in vivo* tests (Figure S2).

### Modification of samples

The Ti and PEEK materials were washed in acetone, absolute ethanol and deionized water through ultrasonication for 30 min each. At present, one of the most representative surface treatment methods for the implants is sandblasting followed by acid etching (SLA) [5]. Therefore, the SLA method was employed to modify the surface of pure titanium. Initially, the titanium samples were sandblasted with 60-mesh white corundum (Al_2_O_3_) particles for 30 s. Subsequently, these samples were acid-etched in 4% hydrofluoric acid for 60 s and then ultrasonically cleaned. The resulting samples were labeled as the Ti group.

The PEEK samples were submerged in the concentrated sulfuric acid solution under ultrasonication for 2 min at 25°C to form uniform porous surfaces [19, 34]. Then, the sulfonated PEEK (sPEEK) was ultrasonically rinsed three times with acetone, absolute ethanol and deionized water to eliminate any remains in the pores. Subsequently, the co-decorated sPEEK was fabricated via the hydrothermal method, where the sPEEK was soaked in the 0.01 mol/l Co(OH)_2_ solution and put in an autoclave at 180°C for 6 h. The samples prepared by this method were named the sPEEK-Co group. Dopamine was mixed with the Tris-HCl buffer solution, resulting in a dopamine solution with a 2-mg/ml concentration, and the above sPEEK sheets were soaked in the dopamine solution for 12 h at a constant temperature of 37°C [29, 35]. Only the Tris-HCl solution was added to the sPEEK group. The samples after washing were named the sPEEK-DPA group. After that, PTH (1-34) was added to pure water to prepare the PTH solutions with different concentrations (2, 10, 50 and 100 μg/ml), and the PTH solutions were added to the sPEEK-DPA and sPEEK-Co-DPA at 4°C for 24 h. The materials produced using this method were labeled as the sPEEK-PTH and sPEEK-Co-PTH groups, respectively. Additionally, the samples were named sPEEK-Co-PTH2, sPEEK-Co-PTH10, sPEEK-Co-PTH50 and sPEEK-Co-PTH100, respectively, according to the PTH (1-34) concentration. All samples were sterilized through exposure to ultraviolet (UV) light for 1 h per surface.

In summary, the samples were divided into five groups: Ti, sPEEK, sPEEK-Co, sPEEK-PTH and sPEEK-Co-PTH groups.

### Characterizations of samples

Field-emission scanning electronic microscopy (FE-SEM, JSM-7610 F, JEOL, Japan) was used to observe the microstructures of sPEEK, sPEEK-Co, sPEEK-PTH, sPEEK-Co-PTH and Ti groups. Atomic force microscopy (AFM, BioScope Resolve, Bruker, Germany) was employed to study the surface morphology and surface roughness of samples. The contact angle (CA) instrument (DSA100S, KRUSS, Germany) was exploited to detect the CA to assess the hydrophilicity of each group. The elemental compositions of the samples were analyzed through X-ray photoelectron spectroscopy (XPS, Thermo Fisher Scientific K-Alpha, USA). Fourier-transformed infrared spectroscopy (FT-IR, Bruker Optik, Germany) was adopted to detect chemical functional groups on the sample surface. Inductively coupled plasma mass spectrometry (ICP-MS, NEXION 350×, PerkinElmer) assessed the release of Co ions in the phosphate-buffered saline (PBS) solution for sPEEK-Co and sPEEK-Co-PTH surfaces. Based on prior research [5], the samples with a size of Φ20 × 2 mm^3^ were immersed in 5 ml of PBS solution and cultured at 37°C for 0, 1, 2, 3, 4, 5, 6, 7 and 8 days. Subsequently, 2 ml of supernatant was collected at each time point to detect the concentration of Co ions in the solution. In addition, it should be elucidated that the optimal utilization concentration of PTH solutions was determined based on the subsequent experimental results of cell counting kit-8. As detailed in ‘Cytocompatibility assessment’ section, the optimal concentration of PTH was found to be 50 μg/ml. Thereby, 50 μg/ml of PTH was used to modify the sPEEK-PTH and sPEEK-Co-PTH samples for surface characterization and subsequent related experiments.

### Mechanical properties

Following the ISO 178:2019 standard, the universal material testing machine (UTM 4203, Sunstest Co., Ltd, China) was used to perform three-point bending experiments to evaluate the impact of the coating on the overall mechanical properties of the material substrate. The machine applied a load force of 0–5 kN on the materials with a size of 80 × 10 × 4 mm^3^, a loading speed of 2 mm/min, and a support span of 64 mm. The loading edge and supports of the three-point bending experiment fixture each had a radius of 5 mm. The elastic modulus (*E*, GPa) and flexural strength (*F*_M_, MPa) were determined using the following formulas:

$$E=FL^{3}/4bsh^{3}\;(1-1)$$

$$F_{M}=3FL/2bh^{2}\left(1-2\right)$$

In the formulas, *F* indicates the applied force (N), *L* indicates the span (mm), *b* indicates the materials’ width (mm), *s* indicates the materials’ deflection (mm) and *h* indicates the materials’ thickness (mm).

In addition, the elastic modulus and hardness of the sample surface were detected via the nano-mechanical test system (TI 980, Bruker Hysitron, Germany) by the nanoindentation technology. The applied load range was 0–5000 μN. We read the above results from the corresponding analysis software. The adhesion–tension test was employed to determine the binding strength between the sample coating and the substrate by the UTM at a loading speed of 2 mm/min until fracture happened.

### *In vitro* cell assays

#### Cell culture

All animal operations were approved by the Ethics Committee of Shandong University Stomatology Hospital. Rat bone marrow stem cells (rBMSCs) were acquired from bone marrows of male Sprague Dawley rats (14–21 days old) and cultured as stated before [36]. Shortly, the rats were euthanized, and bilateral femoral bone marrow was flushed out with alpha-minimal essential medium (α-MEM, VivaCell BIOSCIENCES, China) added with 10% fetal bovine serum (Biological Industries, Israel). We changed the medium every third day and utilized the cells at passages 3–6 for subsequent tests. In addition, human umbilical vein endothelial cells (HUVECs) were obtained from the ATCC cell bank and incubated in the endothelial cell medium (ScienCell) in an incubator at 37°C. These cells mentioned above were spread onto different materials at a definite concentration when cells reached 80–90% confluence in the cell culture dish.

#### Flow cytometry identification of BMSCs

The BMSC surface protein expression was detected using the flow cytometer (BD Accuri™ C6 Plus, USA). Then, cells were trypsinized and resuspended to an approximate concentration of 10^6^ cells/ml. We added antibodies to the cell suspensions that served as experimental groups: CD44 (Biolegend), CD45 (BD), CD90 (BD) and CD31 (BD). Cells without antibodies acted as controls. After co-incubation for 30 min in darkness, the cells were cleaned using PBS and resuspended for the flow cytometer detection.

#### Cytocompatibility assessment

The cytocompatibility of all samples was assessed by the cell counting kit-8 and live/dead cell staining assay. According to the instructions of the manufacturer, the proliferation of BMSCs in an amount of 8000 on each sample was assessed in 48-well plates by the cell counting kit-8 (CCK-8, Biosharp, China). In brief, at first, third and fifth days, the samples were washed lightly with PBS, and then the CCK-8 solution was added at a concentration of 100 μl/ml to each well. After the 2-h incubation, 100 μl of the supernatant from each well was diverted into a 96-well plate, and the optical density (OD) values at the wavelength of 450 nm were detected by the microplate reader (BioTek Synergy H1, USA). Live/dead cell staining was conducted to evaluate the viability of 8000 rBMSCs on each sample using a live/dead cell staining kit (Solarbio, China). After 3 days, the samples were rinsed using the PBS solution, then stained with 5 μM calcein-AM (live cells, green fluorescence) and 15 μM PI (dead cells, red fluorescence) for 5 min at 37°C in an incubator in the dark and imaged with a confocal reflection microscope (Leica-DMi8, Germany).

### *In vitro* osteogenic differentiation capacity

After incubation for 1 day, the normal cell culture medium, as described in ‘Cell culture’ section, was substituted with an osteogenic induction medium containing α-MEM with 10% fetal bovine serum, 50 mg/l ascorbic acid, 10 mM β-glycerol phosphate and 10^−8 mol/l^ dexamethasone.

#### Cytoskeleton and protein immunofluorescence staining

After being cultured for 7 days, rBMSCs on samples were fixed using 4% paraformaldehyde and permeabilized with 0.1% Triton X-100 (Solarbio) for 5 min. Next, after being blocked with 10% goat serum (Beyotime, China) for 1 h, the cells were incubated with a rabbit anti-ALP primary antibody (1:50 dilution; HuaBio) overnight at 4°C. It was followed by incubating with CoraLite 594-conjugated goat anti-rabbit IgG (1:300 dilution; Proteintech, China) for 60 min at room temperature. Finally, the BMSCs were stained using FITC-labeled phalloidin (1:200 dilution; Solarbio) for 40 min and DAPI (Solarbio) for 5 min. The micrographs of BMSCs were visualized under confocal laser scanning microscopy (CLSM, Zeiss LSM800) with consistent conditions. The immunofluorescence intensity of each group was semi-quantified by Image J software.

#### Staining and quantification of ALP and ARS

The ALP distribution on samples was revealed by a 5-bromo-4-chloro-3-indolyl phosphate/Nitro blue tetrazolium (BCIP/NBT) ALP color development kit (Beyotime, China). Shortly, the BMSCs were seeded onto samples in 12-well plates (10^5^ cells/well) and cultured for 7 and 14 days. Afterward, BMSCs were fixed with 4% paraformaldehyde for 30 min, and the samples were incubated with the previously prepared BCIP/NBT staining solution for 60 min to stain ALP. Finally, the ALP staining was scanned via a scanner. In addition, for quantitative analysis of ALP activity, BMSCs on the samples were lysed with 1% v/v Triton X-100 (Solarbio) after 14 days of incubation in the culture medium. Subsequently, the protein concentration was measured using the Bicinchoninic Acid (BCA) protein assay kit (Solarbio). Next, the cell lysates were mixed with the working solution of the ALP assay kit (Nanjing Jiancheng Bioengineering Institute, China) and incubated for 15 min. The ALP activity was determined by the microplate reader at 520 nm.

The cellular calcified deposition was evaluated using Alizarin Red S (ARS) staining. In brief, BMSCs on each sample were fixed in 4% formaldehyde for 30 min on Day 14, and next were stained by the 0.2% ARS solution (Solarbio) for 30 min. The pictures of mineralized nodules were obtained via a scanner. Moreover, for quantitative assay, the materials were put into 1 w/v% hexadecylpyridinium chloride for 30 min. Next, 100 μl supernatant was moved into 96-well plates, and their absorbance values were detected by a microplate reader at 562 nm.

#### Osteogenic-related gene expression

A seeding density of 10^5^ cells per well was applied to the 12-well plates. The quantitative reverse transcription-polymerase chain reaction (qRT-PCR) was employed for detecting the gene expression levels of ALP, Runx2, osteopontin (OPN) and collagen-1 (COL-1). It also evaluated the bone-improving influence of samples on BMSCs after 7 and 14 days. The experiment was repeated three times, and the 2^–ΔΔCt^ way was utilized for quantitative assessment. The sequences of primers for each gene are depicted in Table S1.

### *In vitro* angiogenic ability

#### Tubule formation test of HUVECs

Vascularized tests of Matrigel (Mogengel-Bio) were utilized to assess the tubule formation capacity of HUVECs on samples. To sum up, preceding the test, 60 μl of Matrigel that had been pre-chilled was covered onto 96-well plates at 37°C for 30 min. Then, the HUVECs (2 × 10^4^ cells per well) cultured on different materials for 4 days were trypsinized and subsequently seeded on the Matrigel-covered 96-well plates. After a 6-h incubation at 37°C, the Matrigel cultures were visually documented by the optical microscope (OLYMPUS IX73, Japan), and the parameters of vascularization, including the number of meshes, segments, nodes and total length, were evaluated using Image J software.

#### Angiogenic-related gene expression

The RT-qPCR was employed for studying the gene expression levels of HIF-1**α**, VEGF, basic fibroblast growth factor (bFGF) and stem cell growth factor (SCF), and to assess the angiogenic influence of samples on HUVECs after 2 and 4 days. A seeding density of 10^5^ cells per well was applied to the 12-well plates, and the sequences of primers for each gene are depicted in Table S1.

### *In vitro* antibacterial ability

#### Bacterial live/dead staining and morphology

In this study, the antibacterial tests were conducted using *S. aureus* and *E. coli*. After sterilization, samples of each group were loaded into centrifuge tubes, with each tube containing 5 ml of bacterial suspension at a concentration of 10^6^ CFU/ml. Subsequently, these tubes were incubated in an aerobic bacterial culture chamber at 37°C for 12 h, after which bacterial cultures were collected.

For live/dead bacterial detection, 100 μl of a live/dead bacterial staining solution (ABP Biosciences) was used to resuspend the bacterial cultures. Bacterial states were observed using a fluorescence microscope (OLYMPUS IX73, Japan). A portion of the abovementioned bacterial cultures were resuspended using 4% paraformaldehyde (Biosharp, China) at 4°C for 12 h. Following this, a range of ethanol solutions at 30%, 55%, 75%, 85%, 95% and 100% concentrations were used for gradient dehydrating of the bacteria, each step lasting for 15 min. Subsequently, the bacteria were gained via centrifugation at 8000 rpm for 8 min. The gained bacteria were then resuspended in anhydrous ethanol for scanning electron microscopy (SEM) evaluation of bacterial morphology.

#### Colony counting assay

*E. coli* and *S. aureus* were co-cultured at a concentration of 10^6^ CFU/ml in a volume of 300 μl with the samples in centrifuge tubes for 12 h. For the bacterial adhesion test, the materials underwent three washes with the sterile PBS solution. Then, they were placed in another sterile centrifuge tube, and surface-adhered bacteria were washed out using ultrasonication (2 min, 300 W) and collected before being diluted by the PBS solution. Subsequently, they were spread on sterile nutrient agar plates for 18 h. The photos of colonies were captured by a digital camera (Canon), and the number of colonies was measured by Image J.

### *In vivo* animal assays

All animal care and assays complied with international standards and were approved by the Ethics Committee of Shandong University Stomatology Hospital. In the experiments, 25 male SD rats (3 months old, each comprising five rats) were used and then randomly allocated into five groups: sPEEK, sPEEK-Co, sPEEK-PTH, sPEEK-Co-PTH and Ti groups. Before the surgical implantation, the rats were anesthetized using the 4% pentobarbital sodium (40 mg/kg) administered via intraperitoneal injection. The surgical procedures were performed in aseptic circumstances. The rat skin at the distal end of the femur was incised, and the muscles were directly incised to disclose the femoral condyle. Subsequently, a hole with a diameter of 2 mm was created in the distal femur by vertically employing a dental drill until the hole depth reached 7 mm. The samples were positioned within the bone defects on both sides of the femur. Following this, the incisions were sutured, and all rats received a 3-day course of muscle injections of antibiotics. In the fourth week after implantation, the rats were humanely euthanized, and the femurs, along with the implants, were collected. The soft tissue surrounding the femurs was removed, and the specimens were fixed in 4% paraformaldehyde for a minimum of 48 h before conducting further assessments.

#### micro-CT assessment

The live animal computed tomography system (Quantum GX2, PerkinElmer, Japan) was employed to scan the femurs, and 3D reconstruction was carried out by the corresponding software. The region of interest (ROI) was considered an annular domain encompassing 200 μm of tissue around the implant to facilitate the assessment of osseointegration surrounding the implant. In addition, the evaluation of osteogenesis involved measuring parameters such as bone-to-tissue volume ratio (BV/TV), trabecular number (Tb.N) and trabecular thickness (Tb.Th).

#### Histological assessment

After cleaning with flowing water, the tissue samples underwent decalcification using a 10% EDTA solution for 8 weeks, allowing for the effortless insertion of a needle without resistance. The implants were subsequently extracted cautiously and aligned with the bone axis to avoid damage to the surrounding tissues. Following this, the specimens underwent a series of procedures, including ethanol gradient dehydration, xylene transparency, paraffin embedding and transverse sectioning into 5 μm slices around the implant sites. Subsequent assessments included hematoxylin and eosin (H&E) staining, along with modified Masson staining, to evaluate the development and the maturity of the new bone tissue successively. Moreover, the decalcified sections were subjected to immunohistochemical staining to observe the expression of p-PI3K (CST, USA) and p-AKT (CST, USA) within the bone tissue surrounding the implants. These sections were visualized by an optical microscope (OLYMPUS BX51, Japan), and Image J software was employed to analyze every section for quantitative analysis.

#### Biosafety and biotoxicity

After euthanasia, the pertinent organ tissues (such as the heart, liver, spleen, lung and kidney) were harvested and prepared for H&E staining to evaluate the systemic biosafety and biotoxicity of the implants. In the H&E staining process, the nuclei of cells were stained using hematoxylin (Solarbio), and eosin (Solarbio) staining was for cytoplasm and extracellular matrix (ECM).

### Statistical analysis

Statistical analysis was conducted by SPSS 23.0. One-way ANOVA was employed to compare the data, and the findings were expressed as the mean ± standard deviation (SD) based on at least three distinct measurements. Statistical significance was ascertained for *P*-values below 0.05.

## Results

### Characterizations of samples

**Figure 1.**
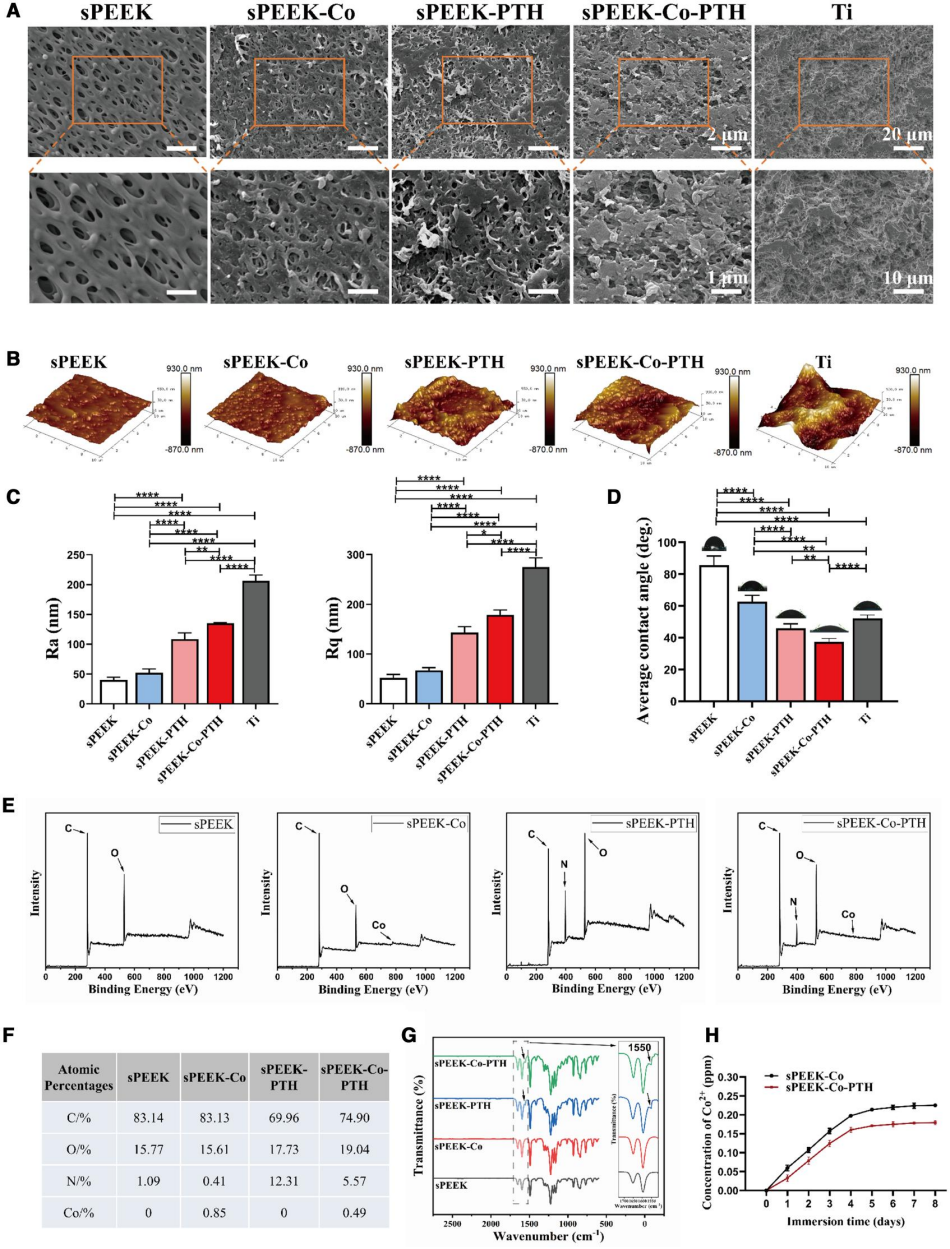
Characterization of samples. (**A**) FE-SEM images of sPEEK, sPEEK-Co, sPEEK-PTH, sPEEK-Co-PTH and Ti at low (upper row) and high (lower row) magnifications. (**B**) AFM images of sPEEK, sPEEK-Co, sPEEK-PTH, sPEEK-Co-PTH and Ti. (**C**) AFM corresponding Ra and Rq analysis results (*n* = 3). (**D**) Water CAs on the surface of sPEEK, sPEEK-Co, sPEEK-PTH, sPEEK-Co-PTH and Ti (*n* = 5). (**E**) XPS spectra results of each group. (**F**) The atomic percentages of carbon, oxygen, nitrogen and cobalt through XPS analysis. (**G**) The FTIR spectra results, and the solid line box within is a magnified view of the dashed line box. (**H**) Cumulative Co ions concentrations released from sPEEK-Co and sPEEK-Co-PTH materials in PBS solution (*n* = 3).

Figures S1 and S2 depict the physical pictures of the samples employed in cell and animal experiments, respectively. The surface morphology of the materials was assessed using FE-SEM and AFM techniques. SEM images (Figure 1A) revealed the formation of a 3D porous topological structure on PEEK substrates post-treatment with concentrated sulfuric acid. Following hydrothermal processing in a Co(OH)_2_ solution, the surfaces of the sPEEK-Co samples exhibited uniformly distributed small particles and wrinkles. We speculated that the observed wrinkling might be attributed to the high temperature and pressure conditions experienced during processing. AFM analysis (Figure 1B and C) indicated a slight increase in the surface roughness of the sPEEK-Co group (Ra = 52.07 ± 6.47 nm, Rq = 67.17 ± 5.63 nm) compared to the sPEEK group. However, no statistical significance was observed in the contrast. Furthermore, surface modifications involving dopamine and PTH (1-34) protein coatings led to partial coverage of the porous structure in sPEEK-PTH and sPEEK-Co-PTH samples (Figure 1A). Due to the dopamine and PTH coatings, the surface roughness of samples all had a notable increase, positively affecting hydrophilicity and promoting cell adhesion of samples. However, the roughness was still not as good as the Ti group (Ra = 206.70 ± 9.50 nm, Rq = 275.00 ± 18.36 nm). The hydrophilicity of each sample surface was measured through water CA testing (Figure 1D). The results were as follows: sPEEK exhibited a water CA of 85.72 ± 5.68°, significantly higher than the Ti group (52.06 ± 2.18°). After hydrothermal processing, the water CA of sPEEK-Co decreased to 62.81 ± 3.87°, which can be attributed to the increased hydrophilicity induced by Co ions. After the deposition of dopamine and PTH (sPEEK-PTH and sPEEK-Co-PTH), a notable reduction in the water CA was noticed, where the CA of the sPEEK-Co-PTH group was even lower than that of the Ti group (approximately 15°).

XPS measurements were carried out to analyze the surface chemical compositions of each sample, as illustrated in Figure 1E and F. For the sPEEK samples, carbon and oxygen peaks were observed as the predominant atomic elements. However, the surfaces of sPEEK-Co and sPEEK-Co-PTH samples had clear cobalt (Co) peaks, signifying the effective incorporation of Co elements onto the sample surfaces. PTH-coated samples (sPEEK-PTH and sPEEK-Co-PTH) exhibited distinct nitrogen (N) peaks, confirming the successful surface modification with polydopamine-adhesive PTH. Figure S3 shows the high-resolution N 1 s XPS spectra of the sPEEK-PTH and sPEEK-Co-PTH groups, both of which had obvious N 1 s peaks around 400 eV, likely originating from the -N-H bonds in polydopamine and PTH (1-34). Figure S4 shows the high-resolution N 1 s XPS spectra of the sPEEK and sPEEK-Co groups, but these N peaks were not particularly obvious. In addition, Figure 1F presents the atomic percentages of carbon, oxygen, nitrogen, cobalt through XPS analysis. The sPEEK and sPEEK-Co groups had trace amounts of N elements, which may be due to the adsorption of nitrogen when the samples were exposed to the air (Figure 1F). And the Co content on sPEEK-Co-PTH was 0.49 at%, lower than the 0.85 at% Co content on sPEEK-Co. This difference in cobalt content may be attributed to the deposition of dopamine on the sample surface, which covered the cobalt element, resulting in the reduction in cobalt content on the surface of sPEEK-Co-PTH. Compared to sPEEK, new cobalt elements were found on the sPEEK-Co and sPEEK-Co-PTH surfaces, while obvious N peaks were observed on sPEEK-PTH and sPEEK-Co-PTH surfaces. Comparable outcomes were also observed in the FTIR spectra (Figure 1G). In contrast to the sPEEK and sPEEK-co groups, the surfaces of sPEEK-PTH and sPEEK-Co-PTH groups exhibited a stretching vibration peak at ∼1550 cm^−1^, corresponding to ‘-CO-NH-’ (peptide bond). This signifies that the PTH was effectively attached to the sPEEK surface. Figure 1H depicts the release kinetics of Co^2+^ into the PBS solution over an 8-day period. Over 8 days, both sPEEK-Co and sPEEK-Co-PTH samples displayed a comparable tendency in the release of Co ions, with the release rate gradually stabilizing from the third day onwards. For the sPEEK-Co group, the cumulative release of Co ions was 0.225 ppm, while for the sPEEK-Co-PTH group, it was 0.179 ppm. Furthermore, the dopamine overlay on the sPEEK-Co-PTH substrates inhibited the release of Co ions. This resulted in slightly lessened levels of Co ions release from the sPEEK-Co-PTH material at each time point compared to sPEEK-Co. As shown in Figure S5, the surface morphology of the sPEEK-Co and sPEEK-Co-PTH samples exhibited almost no changes after the ion release tests, except for an obvious reduction of small particles.

### Mechanical properties

**Figure 2.**
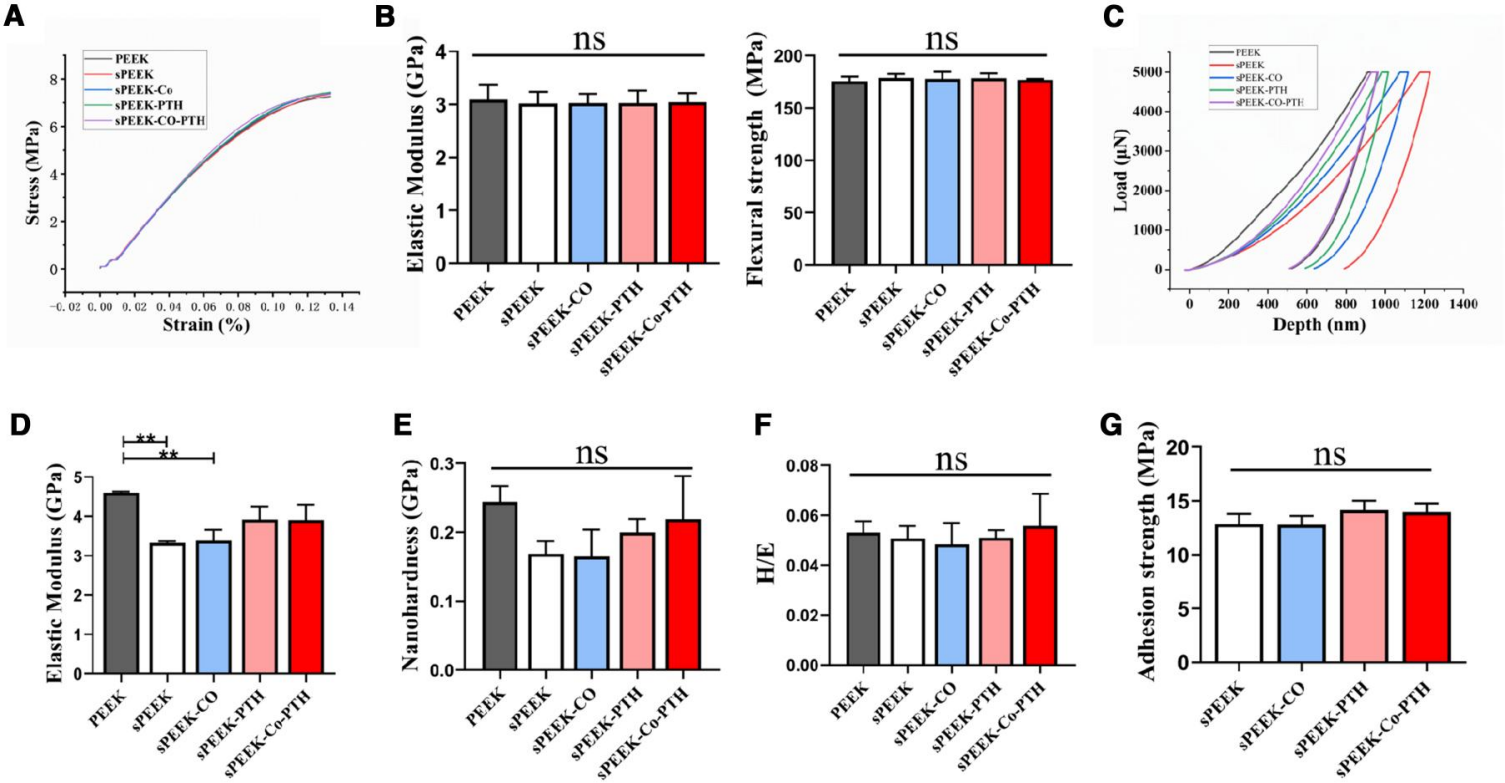
Mechanical properties test of the samples. (**A**) Stress–strain curves of each group after the three-point bending test. (**B**) The elastic modulus and flexural strength of different samples (*n* = 6). (**C**) The load–depth curves of each group after the nano-mechanical test. (**D**) The assessment of elastic modulus of each group (*n* = 3). (**E**) The nano-hardness of each group (*n* = 3). (**F**) The H/E ratio of each group (*n* = 3). (**G**) Adhesion strength between the sample coating and the substrate (*n* = 4). (*P < 0.05, **P < 0.01, ***P < 0.001, and ****P < 0.0001, comparing pairs with each other).

The results of the three-point bending test are illustrated in Figure 2A, where the typical stress-–strain curves for each group are plotted. It was evident that the curves for each group exhibited substantial overlap and displayed a smooth and uninterrupted nature, signifying the absence of material abrupt fracture. Figure 2B shows the elastic modulus of different samples, which was approximately 3.1 GPa, and the flexural strength, approximately 177 MPa. The analysis findings indicated that introducing porous surfaces and surface modifications did not alter the original mechanical strength of PEEK, and no deformation was observed. This suggests that surface-modified PEEK samples retained the original elastic modulus and flexural strength of pure PEEK. No statistically significant differences were observed in these groups, and all exhibited excellent flexural strength.

Figure 2C presents load–depth curves for each group after the nano-mechanical test. Analysis of different curves enabled the assessment of elastic modulus and nano-hardness for each group, as depicted in Figure 2D and E. The results indicated that while the elastic modulus of sPEEK and sPEEK-Co samples exhibited a slight reduction compared to PEEK, the surface elastic modulus of sPEEK-PTH and sPEEK-Co-PTH samples was approximately 4 GPa, with no statistically significant difference compared to PEEK. This may be attributed to dopamine, which enhanced the elastic modulus of the surface. Figure 2E and F demonstrates that the nano-hardness (approximately 0.2 GPa) and the ratio of hardness and elastic modulus (H/E ratio) for surfaces of each group exhibited no statistically significant differences. Additionally, the H/E ratio can be utilized to assess the wear resistance of material coatings, with higher values indicating superior wear resistance [37, 38]. This aligns with the outcomes of the three-point bending experiment, confirming that surface-modified PEEK samples, similar to pure PEEK, all maintained excellent mechanical performance. Figure 2G shows that there was no statistically significant difference in adhesion strength among the groups, with all values around 13 MPa. This suggests that the sample coatings in each group can firmly bond to the substrates.

### Cytocompatibility assessment

**Figure 3.**
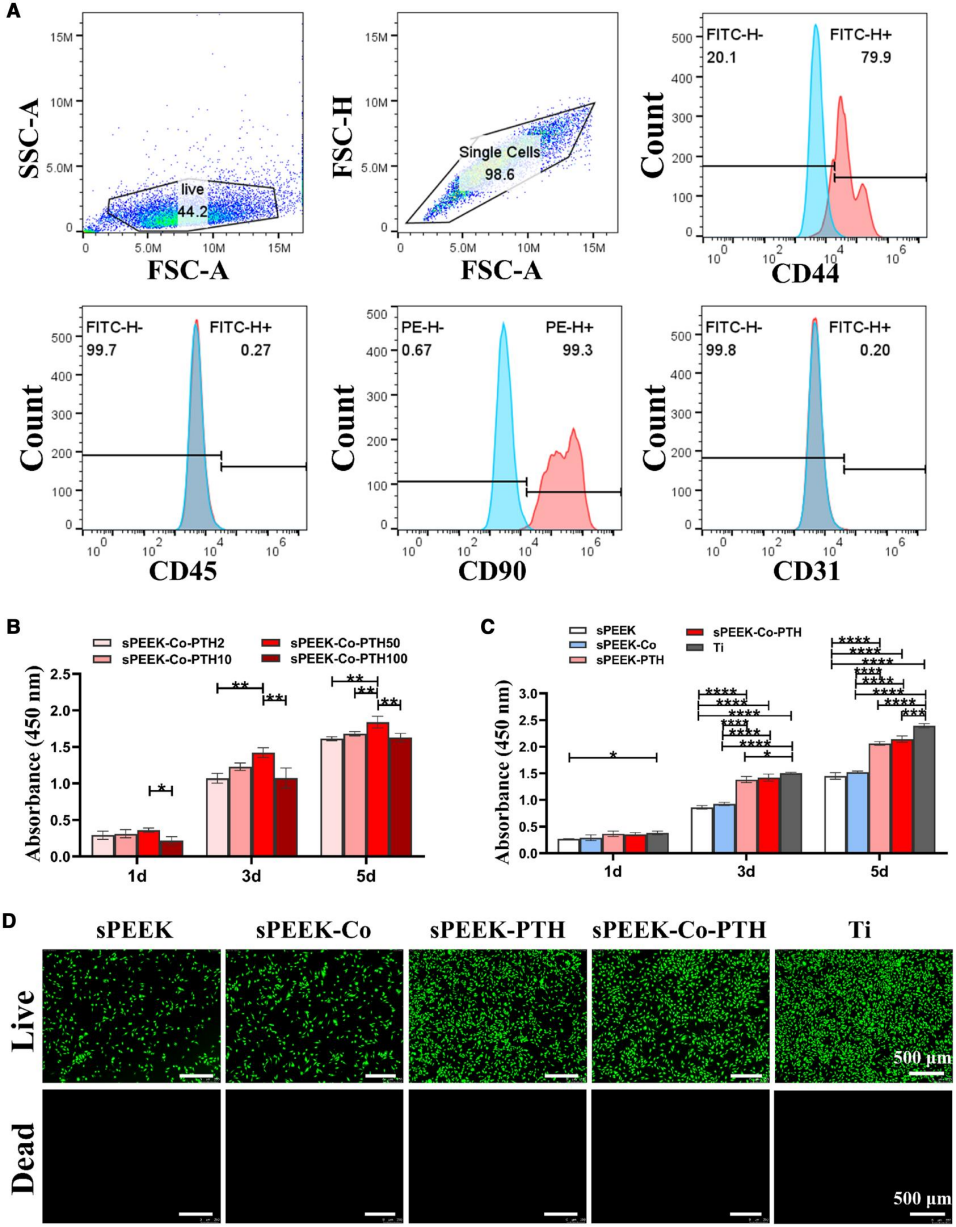
Flow cytometry identification of BMSCs and cytocompatibility assessment of the samples. (**A**) Flow cytometry analysis showed positive surface marker CD44 (79.9%) and CD90 (99.3%), and negative CD45 (0.27%) and CD31 (0.20%). (**B**) CCK-8 results of BMSCs cultured on different samples for 1, 3 and 5 days (*n* = 3). (**C**) CCK-8 results of BMSCs cultured on different samples for 1, 3 and 5 days (*n* = 3). (**D**) The live/dead staining assay of BMSCs on the surfaces of the samples at the third day.

The extracted primary rBMSCs grew adherently in the culture dish, exhibiting a fibroblast-like morphology and swirly and colony-like growth (Figure S7). The third-generation cells were subjected to flow cytometry analysis (Figure 3A). They indicated positive expression of stem cell surface markers CD44 (79.9%) and CD90 (99.3%) in the molecular phenotype. In contrast, non-stem cell-related markers CD45 (0.27%) and CD31 (0.20%) showed negative expression. These findings confirmed that the cells isolated from rat bone marrow were predominantly rBMSCs, consistent with the surface marker criteria established by the International Association for Cell Therapy [39].

BMSCs were seeded on each group of samples to evaluate the *in vitro* cytocompatibility of various samples. Four different concentrations were examined using CCK-8 assays to identify the most suitable co-culture concentration of PTH on sPEEK materials. As illustrated in Figure 3B, the sPEEK-Co-PTH50 group exhibited the most favorable proliferation effect, prompting the selection of the 50 μg/ml PTH concentration for subsequent experiments. Subsequently, the samples were categorized into five groups: sPEEK, sPEEK-Co, sPEEK-PTH, sPEEK-Co-PTH and Ti. The proliferation of BMSCs on each group of materials was assessed using CCK-8 assays. Figure 3C shows that, after 3 and 5 days of culture on different samples, the cell viability in sPEEK-PTH, sPEEK-Co-PTH and Ti groups was significantly higher than the other two groups. Moreover, by the fifth day, the cell viability in the sPEEK-PTH and sPEEK-Co-PTH groups closely followed that of the Ti group. Furthermore, Figure 3D presents the results of live/dead cell staining after 3 days of cell culture, which corroborated the CCK-8 findings. No dead cells were observed on any sample surface, indicating an absence of cytotoxicity. In conclusion, the results suggested a cooperative impact of dopamine and PTH protein in enhancing the proliferation of BMSCs.

### *In vitro* osteogenic differentiation capacity

**Figure 4.**
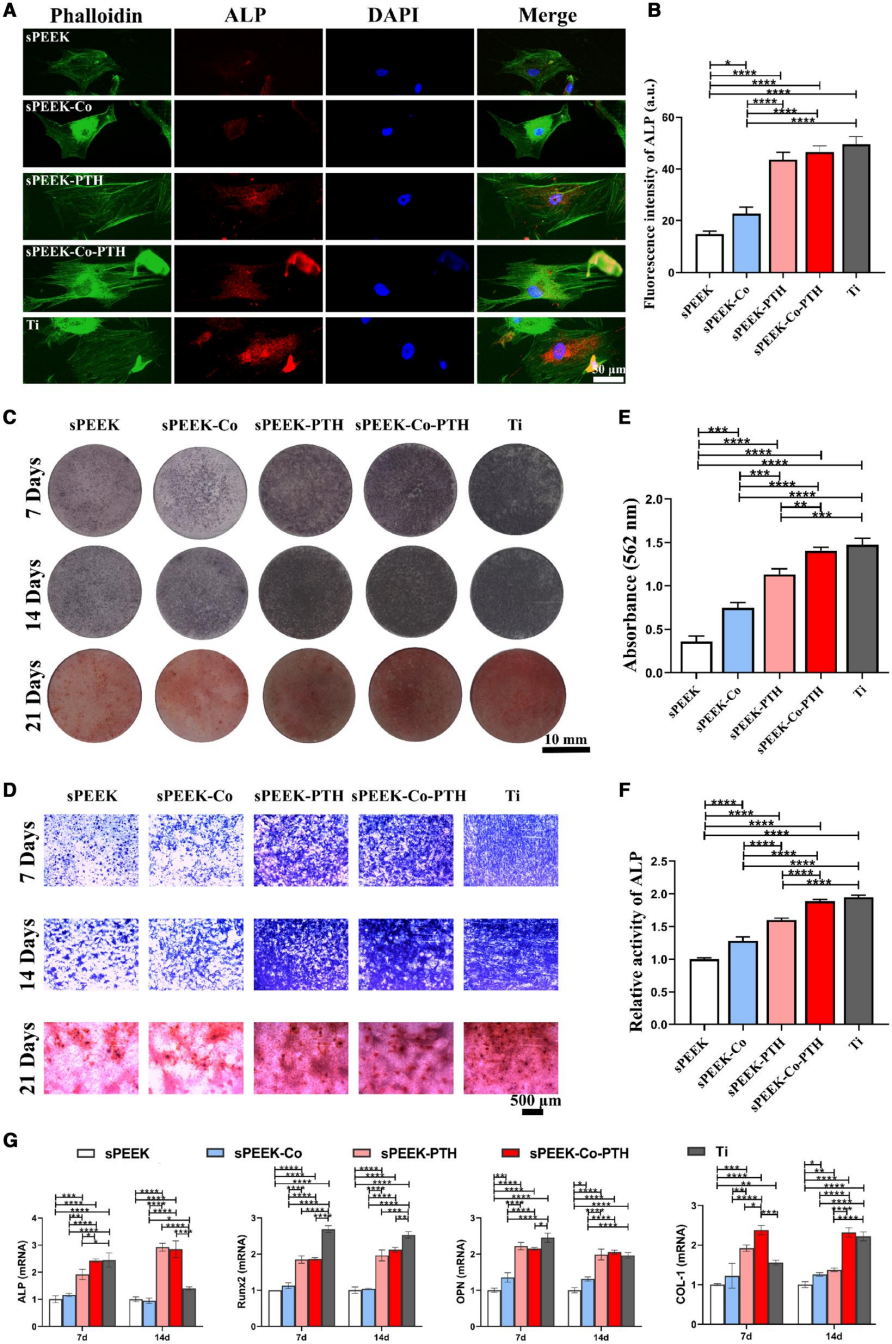
The evaluation of osteogenic differentiation *in vitro*. (**A**) The BMSCs immunofluorescent staining of cytoskeleton, ALP protein and cell nuclei on various material substrates after a 7-day culture period. (**B**) Quantitative analysis of ALP protein fluorescence intensity was conducted (*n* = 3). (**C**) ALP staining of BMSCs cultured for 7 and 14 days and alizarin-red staining of BMSCs cultured for 21 days (the images obtained by a scanner). (**D**) The corresponding pictures under a stereomicroscope. (**E**) Quantitative analysis of alizarin-red staining results (*n* = 3). (**F**) ALP relative activity on Day 14 (*n* = 3). (**G**) The expression of osteogenesis-related genes was estimated by RT-qPCR on Days 7 and 14 (*n* = 3).

ALP immunofluorescence staining was employed to measure the expression of ALP protein in cells cultured on different samples after 7 days. The ALP immunofluorescence staining results (Figure 4A) revealed that cells on sPEEK-PTH, sPEEK-Co-PTH and Ti samples displayed a polygonal morphology with a larger cell spreading region and higher ALP protein expression. In contrast, cells on sPEEK and sPEEK-Co samples displayed a narrower cell morphology with a smaller spreading area, resembling typical spindle shapes and exhibited lower ALP protein expression. Additionally, quantitative analysis of ALP fluorescence intensity (Figure 4B) agreed with the aforementioned ALP staining results, indicating significantly higher ALP protein fluorescence intensity in cells on sPEEK-PTH, sPEEK-Co-PTH and Ti samples compared to sPEEK and sPEEK-Co groups.

ALP staining was conducted on Days 7 and 14 of the co-culture period, as depicted in Figure 4C and D, to further assess the influence of various materials on the osteogenic differentiation of BMSCs. The findings revealed a pronounced elevation in the ALP staining area for samples from the sPEEK-PTH, sPEEK-Co-PTH and Ti groups compared to both sPEEK and sPEEK-Co groups. Moreover, the sPEEK-Co group also exhibited a larger staining area than the sPEEK group. ECM mineralization is a vital marker representing late-stage osteogenic differentiation. Following a 21-day cell culture period, the evaluation of mineralized nodules on material surfaces was carried out through the ARS staining (Figure 4C and D) and the quantitative measurements of calcium deposition (Figure 4E). Similar to the ALP staining trends, ARS staining demonstrated a higher presence of red calcium deposition nodules on samples from the sPEEK-PTH, sPEEK-Co-PTH and Ti groups. The area of the sPEEK-Co-PTH and Ti groups was larger compared to the sPEEK-PTH group. This observation was further substantiated through quantitative measurements (Figure 4E). As depicted in Figure 4F, by Day 14, the relative expression level of ALP was notably highest in the sPEEK-Co-PTH and Ti groups, aligning with the ALP and ARS staining results. These findings underscored the favorable impact of co-loading Co and PTH on the osteogenic differentiation of the materials.

The qRT-PCR was employed to assess the expression of osteogenic-related genes, comprising ALP, Runx2, OPN and Col-1 (Figure 4G), to further investigate osteogenic differentiation potential. In general, the analysis results were consistent with the previous content. BMSCs cultured on sPEEK-PTH, sPEEK-Co-PTH and Ti substrates on Days 7 and 14 exhibited elevated expression levels of osteogenic-related genes, surpassing those of sPEEK and sPEEK-Co groups. Notably, the sPEEK-Co-PTH group demonstrated the highest expression levels of ALP, OPN and Col-1, even comparable to the levels observed in the Ti group. Overall, this indicated that the synergistic impact of Co and PTH on the sPEEK substrate promoted the expression of osteogenic-related genes.

### *In vitro* angiogenic ability

**Figure 5.**
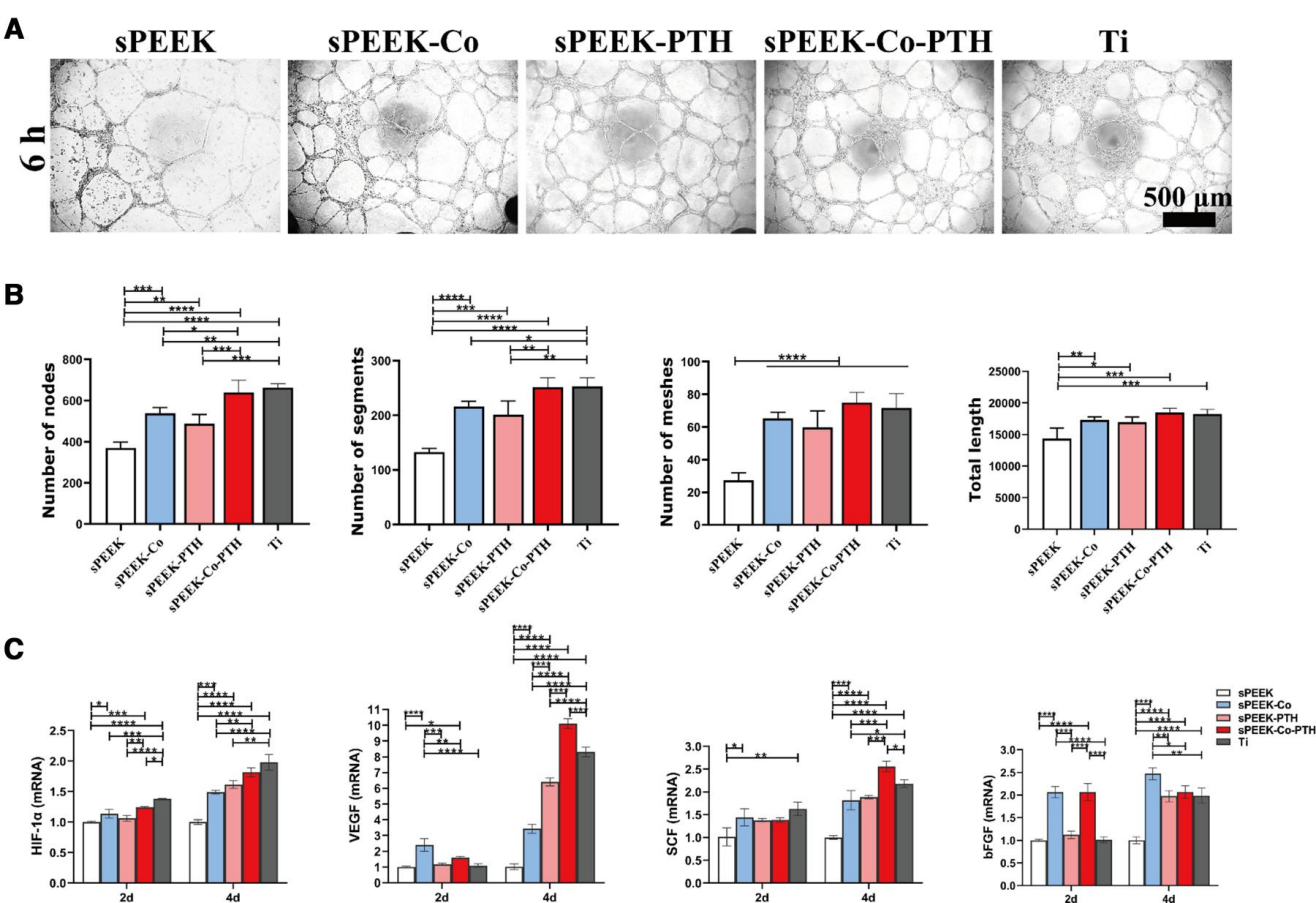
Assessment of angiogenic ability *in vitro*. (**A**) Representative graphs depicting HUVECs tube formation following a 6-h incubation. (**B**) The images illustrating the data related to tube formation parameters, which encompass the number of nodes, segments, meshes and total tube length (*n* = 3). (**C**) RT-qPCR analysis of the expression of angiogenesis-related genes, including HIF-α, VEGF, bFGF and SCF in HUVECs on Days 2 and 4 (*n* = 3).

Figure S8 shows the microscopic pictures of HUVECs, which were arranged like cobblestones and had an epithelial cell-like morphology. HUVECs were cultured on a Matrigel-coated substrate to simulate ECM conditions for angiogenesis. Following a 6-h incubation, sPEEK-Co, sPEEK-PTH, sPEEK-Co-PTH and Ti groups notably induced the formation of more vascular-like structures compared to the sPEEK group (Figure 5A). This was evidenced by increased branch nodes and mesh-like structures. Quantitative analysis of the formed tubular structures, comprising the number of nodes, segments, meshes and total tube length using Image J software (Figure 5B), revealed that the sPEEK group had the lowest values. In contrast, the sPEEK-Co, and sPEEK-PTH groups exhibited intermediate values. Notably, the sPEEK-Co-PTH and Ti groups showed the highest values. These results collectively indicate the significant impact of the Co and PTH co-loading on the angiogenesis of the materials.

The findings from the RT-qPCR analysis (Figure 5C) aligned with the Matrigel tube formation experiment, assessing the expression of angiogenesis-related genes, including HIF-α, VEGF, bFGF and SCF. Notably, the sPEEK-Co group showed notably higher expression of angiogenic genes compared to the sPEEK group. The sPEEK-Co-PTH group demonstrated the highest expression, reaching levels equivalent to the Ti group. This enhanced angiogenic gene expression of the sPEEK-Co-PTH group can likely result from the sustained release of Co ions from the material. The above results unequivocally confirmed the remarkable *in vitro* angiogenic potential of samples in the sPEEK-Co-PTH group.

### *In vitro* antibacterial ability

**Figure 6.**
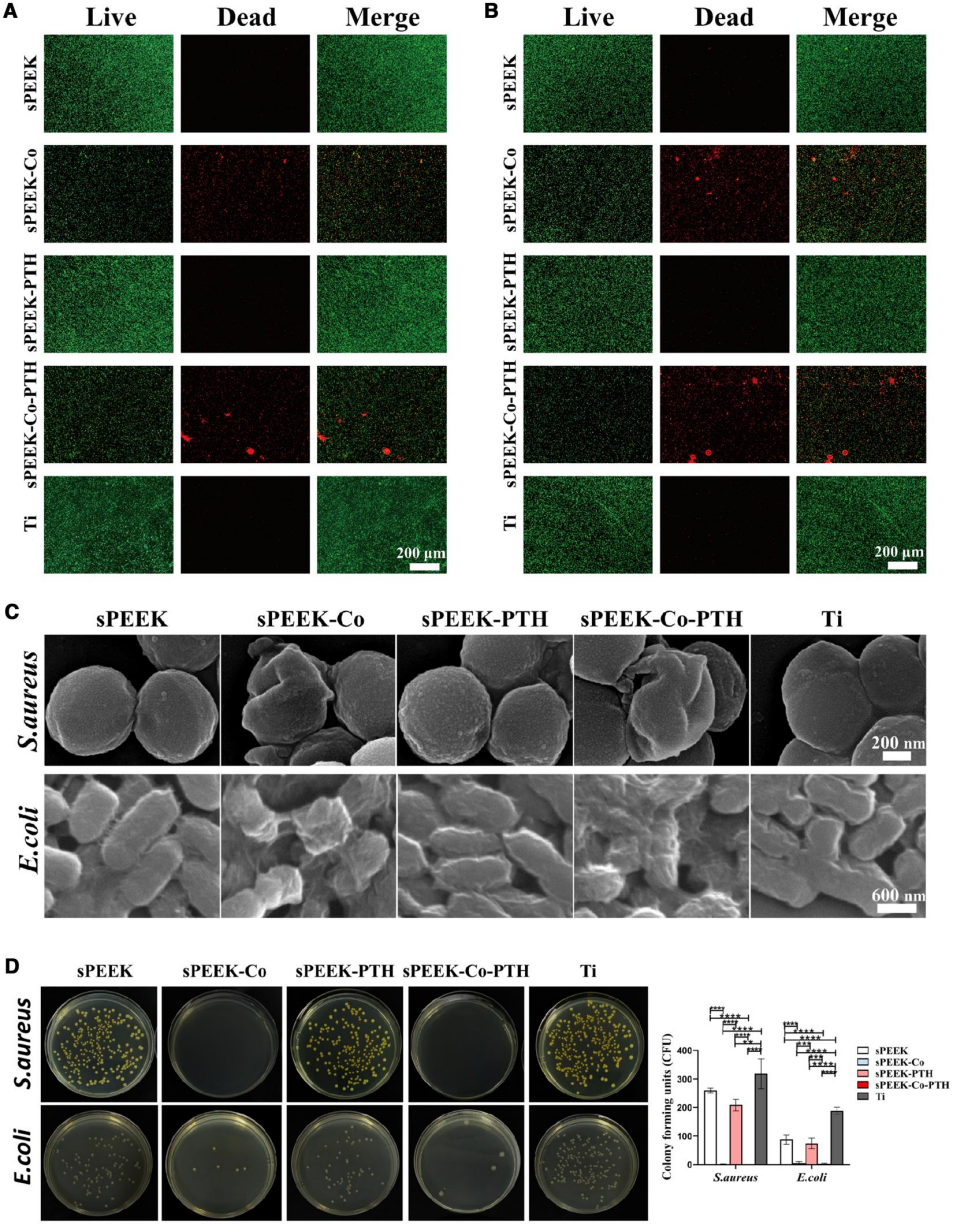
Assessment of antibacterial ability *in vitro*. (**A**) Live/dead fluorescence images of *S. aureus* cultured on different samples for 12 h. (**B**) Live/dead fluorescence images of *E. coli* cultured on different samples for 12 h. (**C**) SEM images of *S. aureus* and *E. coli* cultured on different substrates for 12 h. (**D**) The seeded bacteria were incubated for 18 h and the colonies were counted in the bacterial adhesion test (*n* = 3).

*S. aureus* and *E. coli* are well-known representatives of Gram-positive and Gram-negative bacteria, respectively. Antimicrobial properties are crucial for bone implants. Therefore, we employed *E. coli* and *S. aureus* to study the antimicrobial efficacy of modified materials. Figure 6A and B depicts fluorescence staining pictures of live/dead *S. aureus* and *E. coli* cultured on different samples, respectively. Living bacteria are represented in green, while dead bacteria are shown in red. The results indicated a pronounced reduction in the number of live bacteria in the sPEEK-Co and sPEEK-Co-PTH groups, where dead bacteria were notably obvious. In contrast, the other groups exhibited extensive green fluorescence, and the number of dead bacteria which were stained red was significantly smaller. Figure 6C displays SEM images of *S. aureus* and *E. coli* cultured on different sample surfaces to further confirm the bacterial morphology. SEM analysis revealed significant bacterial shrinkage and deformation in the sPEEK-Co and sPEEK-Co-PTH groups. In contrast, bacteria on the other sample surfaces remained intact, consistent with the results of the bacterial staining. These findings collectively underscore the strong antimicrobial efficacy of the sPEEK-Co and sPEEK-Co-PTH groups against *E. coli* and *S. aureus*, which is possibly attributed to the antimicrobial properties of Co ions.

Figure 6D presents photographs of bacteria retrieved from samples after the bacterial adhesion test, followed by their recultivation on agar plates. The quantitative assessment of bacterial colony counts on each plate was conducted to evaluate the antimicrobial activity of each sample. The small white dots on the plates represent viable bacterial colonies. The results demonstrate that the bacteria on the samples in the sPEEK, sPEEK-PTH and Ti groups formed many large colonies. In contrast, the number of colonies formed on sPEEK, sPEEK-PTH and Ti samples had a significant reduction. In general, the antimicrobial performance of sPEEK-Co and sPEEK-Co-PTH groups may result from the release of Co ions and the reduction in bacterial adhesion to the material surface.

### *In vivo* animal assays

**Figure 7.**
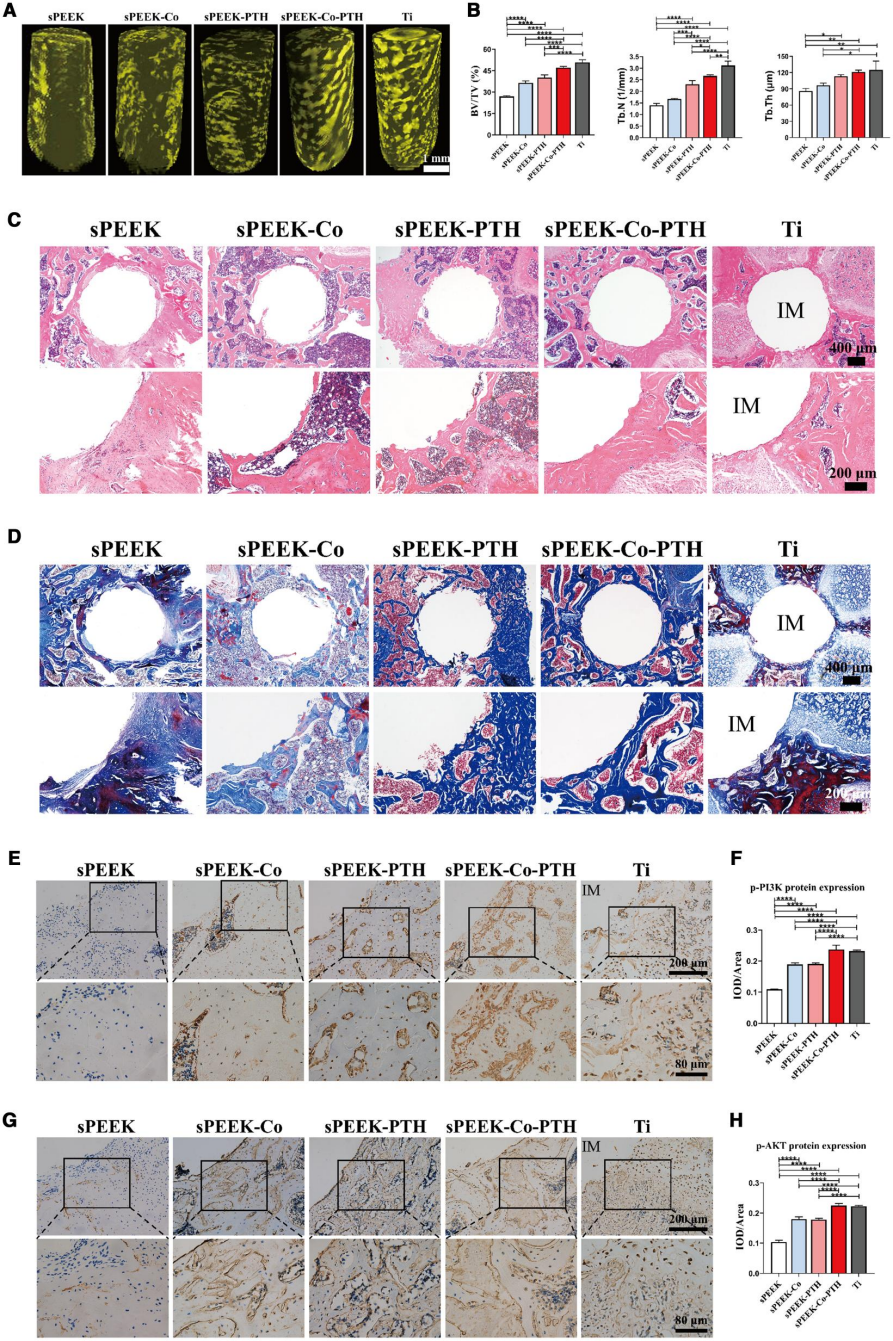
*In vivo* animal assays. (**A**) Reconstructed 3D photographs of femoral implants. (**B**) Quantitative analysis of the micro-CT evaluation of BV/TV, Tb.N and Tb.Th (*n* = 3). (**C**) The images of HE staining, and the magnifications of upper and lower graphs were ×4 and ×10, respectively. (**D**) The images of Masson staining, and the magnifications of upper and lower graphs were ×4 and ×10, respectively. (**E**) IHC graphs for p-PI3K: the magnifications of upper and lower graphs were ×20 and ×40, respectively. (**F**) Quantitative analysis of mean integral optical density (IOD) for p-PI3K (*n* = 3). (**G**) IHC graphs for p-AKT: the magnifications of upper and lower graphs were ×20 and ×40, respectively. (**H**) Quantitative analysis of IOD for p-AKT (*n* = 3). The IM in the image above standing for an implant.

Figure S9 illustrates the entire process of the rat femoral implantation experiment. Micro-CT was employed to reconstruct the microstructure of bone trabeculae surrounding the rat femoral implants. The reconstructed 3D images (Figure 7A) and the quantitative analysis results (Figure 7B) revealed that by the fourth week, the sPEEK-Co-PTH and Ti groups showed nearly complete coverage of trabeculae around the implants. This indicates a favorable osteogenic effect, surpassing that of the sPEEK-PTH group. In contrast, the sPEEK and sPEEK-Co groups displayed relatively fewer trabecular microstructures surrounding the implants. Furthermore, the quantitative analysis results were consistent, and the BV/TV, Tb.N and Tb.Th indices in the sPEEK-Co-PTH and Ti groups were higher than those in the other three groups, with the sPEEK group having the lowest values. The results demonstrate that the sPEEK-Co-PTH material enhanced bone integration around the femoral implants, even comparable to the osteogenic effect of the Ti material.

Tissue staining was conducted to further assess the impact of the various materials on osteogenesis around the femoral implants in rats. H&E staining results (Figure 7C) revealed varying amounts of bone and soft tissue around the implants in all groups. The sPEEK-Co-PTH and Ti groups exhibited the most favorable osteogenic outcomes, while the sPEEK group was predominantly occupied by fibrous tissue. Masson staining results (Figure 7D) indicated that the proportion of mature bone stained in blue and red around the implants was highest in the sPEEK-Co-PTH and Ti groups, significantly exceeding the other three groups. Immunohistochemical staining (IHC) aimed to assess the expression of p-PI3K and p-AKT proteins in the bone area around the implants, and verify possible mechanisms related to bone formation by the material. Figure 7E–H presents the IHC staining and quantitative analysis of p-PI3K and p-AKT in all groups. The findings indicated that the sPEEK-Co-PTH and Ti groups displayed the highest levels of p-PI3K and p-AKT expression around the implants. In contrast, the sPEEK-Co and sPEEK-PTH groups exhibited intermediate expression levels, and the sPEEK group had the lowest expression. Therefore, we speculate that the mechanism of the sPEEK-Co-PTH material in promoting osteogenesis might be linked to the activation of the PI3K/AKT channel. Overall, the combined action of Co and PTH on sPEEK substrates enhanced osteogenic capabilities around the femoral implants in rats.

Figure S10 shows the HE staining of heart, liver, spleen, lung, and kidney tissues in each group. No abnormality or any obvious immune response was detected in the corresponding organs under the microscope, indicating that each group of materials had good *in vivo* biological safety.

## Discussion

As a high-performance polymer material with outstanding biocompatibility and distinctive mechanical characteristics, PEEK has garnered widespread attention. This is due to its exceptional chemical and dimensional stability, an elastic modulus akin to human cancellous bone (3–4 MPa), remarkable frictional performance, radiolucency, low density (1.32 g/cm^3^) and rigidity [40, 41]. Consequently, PEEK is often used as a high-grade substitute for metals in various applications, encompassing medical devices and implants. These advantages make PEEK a promising polymer for hard tissue applications [19, 42]. However, the inherent biological inertness of PEEK contributes to its subpar long-term osseointegration, limiting its broader utilization in bone replacement/repair [19, 29]. Thus, in this study, surface modification of sPEEK was carried out using Co^2+^ and PTH (1-34) to coordinately regulate the vascularization and osteogenic effect of the sPEEK implant. This aimed to improve bone remodeling without compromising its outstanding mechanical properties. Initially, pure PEEK materials were sulfonated by immersion in concentrated sulfuric acid, creating a 3D nano/microstructure on the surfaces [35, 43]. And the 3D structure facilitated the attachment of Co and PTH components and the development of the bone tissues and blood vessels [44]. Sulfonation, a prevalent and effective method to generate porous structures on PEEK, aimed to endow cells with large initial adhesion and spreadability [35, 45]. The resultant pore size on the surface of the material, around 0.1–5.0 μm, was similar to earlier researches that have been reported to facilitate the transmission of biological factors [34, 46]. Subsequently, a hydrothermal reaction of sPEEK in the Co(OH)_2_ solution was employed to bind the substrate with Co ions, further enhancing the surface bioactivity and vascular formation capacity of the material. However, the localized high concentrations of Co ions have been found to adversely affect osteoblast differentiation. According to previous studies [47, 48], the samples were immersed in a 0.01-mol/l Co(OH)_2_ solution for hydrothermal treatment to prepare the sPEEK-Co samples. Previous studies have developed a biokinetic model of cobalt to characterize the dose-response relationship for systemic health effects induced by cobalt [48, 49]. And based on this model, systemic side effects are improbable to occur when the cobalt content is below 0.3 ppm. In this study, the ICP results were within this safety range, and no systemic adverse effects were observed in the histological staining results. ICP and CCK-8 experiments showed the favorable and steady release of Co^2+^ on sPEEK-Co-PTH materials and the presence of polydopamine on the material facilitated the regulation of the Co^2+^ release rate. This steady and sustained release of Co ions is beneficial for angiogenesis and osseointegration activity [25, 26]. Dopamine could form polydopamine via self-polymerization and the polydopamine can exhibit robust adhesion to various substrates [18]. Therefore, PTH (1-34) can adhere firmly to polydopamine on the surface of sPEEK-PTH and sPEEK-Co-PTH samples. The polydopamine-assisted adhesive strategy allowed the deposition of PTH protein on the substrates, enhancing the surface hydrophilicity and cell proliferation of sPEEK and improving its osteogenic and angiogenic capabilities [15, 28]. In addition, based on previous studies [50, 51], the rough surface with high surface energy and hydrophilicity of SLA-treated Ti group could stimulate endothelial cells (ECs) to express angiogenic factor genes and adhesion molecule genes, leading to the formation of high vascular-like structures. Therefore, the Ti group exhibited excellent angiogenic capacity in this study. As widely acknowledged, the proliferation and adhesion of BMSCs are fundamental to *in vivo* bone tissue regeneration [29, 34]. This enhanced hydrophilicity of the sPEEK-Co-PTH group was likely due to the hydrophilic groups, such as the rich amine groups and polar catechol in polydopamine [35, 52] and the abundant amine and carboxyl groups in PTH [53]. Prior researches have indicated that materials with excellent hydrophilicity favor the adhesion and spread of osteoblast cells [35, 54]. Cell morphology and cytoskeletal tension are critical determinants of osteogenic differentiation [55]. The cytoskeleton of BMSCs was visualized through immunofluorescence staining of F-actin to further elucidate the impact of material surfaces on the cell adhesion behavior. The results showed that the sPEEK-Co-PTH group exhibited the maximum cellular spreading area, coupled with excellent biocompatibility, even comparable to the Ti group. Dopamine and PTH on the material, along with the maintained porous structure of the material surface, potentially enhanced osteogenic differentiation by influencing cell morphology and adhesion behavior.

This study fabricated a surface-nanostructured sPEEK-Co-PTH material designed for bone defect repair. It exhibited properties that enhanced vascularization, osteogenic capacity, and antibacterial properties. ALP protein, serving as a pivotal early marker, is utilized for evaluating the osteogenic differentiation degree [56]. Characterization via SEM, XPS and FT-IR confirmed the successful loading of Co elements and PTH onto the surface of sPEEK. The analyses involving CCK8, CLSM, ALP protein staining, matrix mineralization and osteogenesis-related gene expression revealed that loading polydopamine and PTH significantly promoted osteoblast differentiation. And the sPEEK-Co-PTH group demonstrated excellent osteogenic ability, even comparable to the Ti group. Furthermore, *in vivo* tests demonstrated that the surfaces of sPEEK-Co-PTH induced substantial bone tissue growth, facilitating bone regeneration and osseointegration of the implant. The immunohistochemical staining results of paraffin sections in our study further elucidated the potential mechanism by which sPEEK-Co-PTH materials enhanced osteogenesis, possibly involving the activation of the PI3K/AKT pathway. The localized delivery of PTH can favor bone remodeling [57]. Additionally, intermittent low-dose treatment of PTH has been demonstrated to effectively improve osteogenic capacity [58]. Despite the positive effect of intermittent PTH (1-34) administration in enhancing bone formation, its systemic administration may elevate the risk of osteosarcoma [59]. Hence, the local application of PTH emerges as a promising approach, boosting osteogenesis while mitigating the side effects associated with systemic treatment. Numerous investigations have reported superior osseointegration of samples incorporating PTH in treating bone repair compared to samples without PTH [15], consistent with our findings. Prior research has suggested that PTH could participate in bone metabolism through the PI3K/AKT signaling pathway [60]. Additionally, the research by Jorge *et al.* [27] has indicated that cobalt could activate the PI3K/AKT pathway by stimulating ROS production. These results are consistent with our immunohistochemical staining experiments, revealing that the mechanism by which sPEEK-Co-PTH implants promote osteogenesis may be related to activation of the PI3K/AKT pathway by co and PTH. Importantly, the PI3K/AKT pathway is ubiquitously present in cells and participates in the modulation of numerous vital biological processes, particularly in osteoblast proliferation and osteogenic differentiation, as well as osseous generation and remodeling [61, 62]. Vascularization is crucial for facilitating oxygen and nutrient transport during the bone healing and reconstruction process [19]. Nonetheless, direct loading of angiogenic growth factors (SCF, VEGF, etc.) onto samples is not recommended. This is due to their short half-lives and the potential for ectopic vasculature formation [19]. Hence, utilizing divalent ions (Sr^2+^, Co^2+^, Ni^2+^, etc.) to simulate hypoxic microenvironments is an alternative strategy, thereby stimulating VEGF secretion and facilitating vascular regeneration [27]. Results of this study indicated that the presence of Co^2+^ could facilitate vascularization around the implants and the expression of angiogenesis-related genes, thereby improving osteogenic performance, which matched the findings of earlier researches [25, 26]. The widely recognized mechanism behind Cobalt ions promoting angiogenesis involves its ability to simulate hypoxic conditions by stabilizing HIF-1α, thereby upregulating HIF-1α and VEGF expressions linked to neovascular formation [27], and our study aligns with this finding. Regarding antimicrobial therapy, the critical period is the early postoperative period. This article confirmed the antibacterial effect of Co^2+^. This could be associated with the electrostatic attraction between positively charged Co ions and negatively charged bacterial membranes or the production of reactive oxygen species (ROS), leading to membrane permeability damage and bacterial disintegration [63], thereby preventing implant-related infection. The sPEEK implants co-modified with Co/PTH exhibiting excellent osteogenic, angiogenic and antibacterial capabilities, may hold promising potential for dental or orthopedic applications. In conclusion, the nanostructured Co/PTH coating on sPEEK implants could provide a favorable microenvironment for bone regeneration, serving as foundational evidence for their potential use as dental or orthopedic implant materials. However, the osteogenic mechanism of the materials and the long-term efficacy of the implants needs to be verified by further relevant experiments.

## Conclusions

In this study, a combination method involving hydrothermal treatment and polydopamine-aided coating was implemented to devise a multifunctional porous PEEK implant with the Co element and PTH (1-34) protein co-functionalized coatings, denoted as sPEEK-Co-PTH. This was designed to facilitate the vascularization processes and bone formation. We demonstrated that loading biologically active ingredients (Co^2+^ and PTH) onto nanostructured sPEEK significantly enhanced the cellular responses on the substrate. These enhancements included surface hydrophilicity, cell adhesion, cell proliferation, ALP protein staining, calcium deposition, expression levels of osteogenic and angiogenic genes, and the antibacterial properties of the material surface. This ultimately enhanced the biocompatibility of the bioinert implants and promoted osseointegration. Notably, the mechanical properties of the modified materials did not change. Furthermore, our observations suggested that the beneficial osteogenic effects of sPEEK-Co-PTH materials were associated with activating the PI3K/AKT pathway. In conclusion, this research offered a hopeful new approach for the dental and orthopedic fields and may broaden the clinical application scope of sPEEK materials.

## Supplementary Material

rbae067_Supplementary_Data

## Data Availability

Data will be made available on request.
