# Supplementary material for: Multifunctional surface of the nano-morphic PEEK implant with enhanced angiogenic, osteogenic and antibacterial properties
Source: Regen Biomater. 2024 Jun 17;11:rbae067. doi: 10.1093/rb/rbae067 (PMC11226884; doi:10.1093/rb/rbae067)
Supplement: rbae067_Supplementary_Data [file rbae067_supplementary_data.pdf]

Supporting Information

Multifunctional surface of the nano-morphic PEEK implant with enhanced angiogenic, osteogenic, and antibacterial properties

Jiajia Zhang, Tongtong Ma, Xueye Liu, Xiaoran Zhang, Wenqing Meng, Junling Wu *

Department of Prosthodontics, School and Hospital of Stomatology, Cheeloo College of Medicine, Shandong University & Shandong Key Laboratory of Oral Tissue Regeneration & Shandong Engineering Research Center of Dental Materials and Oral Tissue Regeneration & Shandong Provincial Clinical Research Center for Oral Diseases, No.44-1 Wenhua Road West, 250012, Jinan, Shandong, China

*Corresponding author: Junling Wu, doctorwujunling@163.com

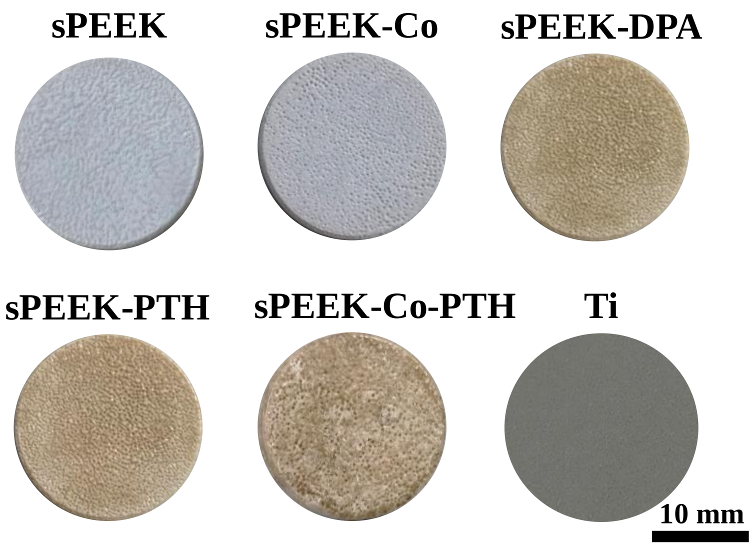

Figure S1: The physical pictures of the samples *in vitro* experiments.

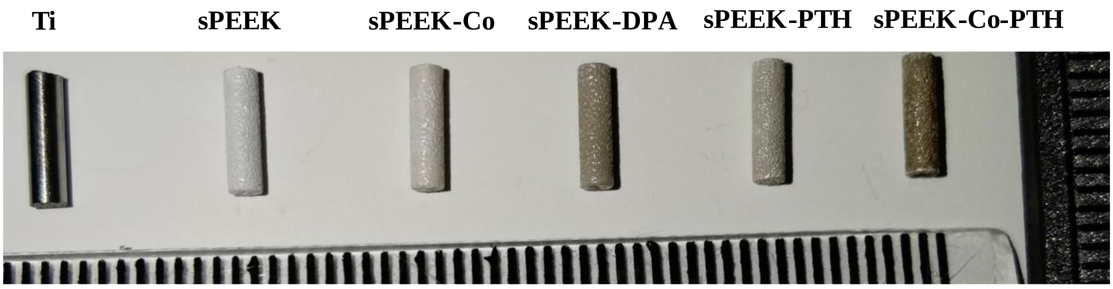

Figure S2: The physical pictures of the femoral implants *in vivo* experiments.

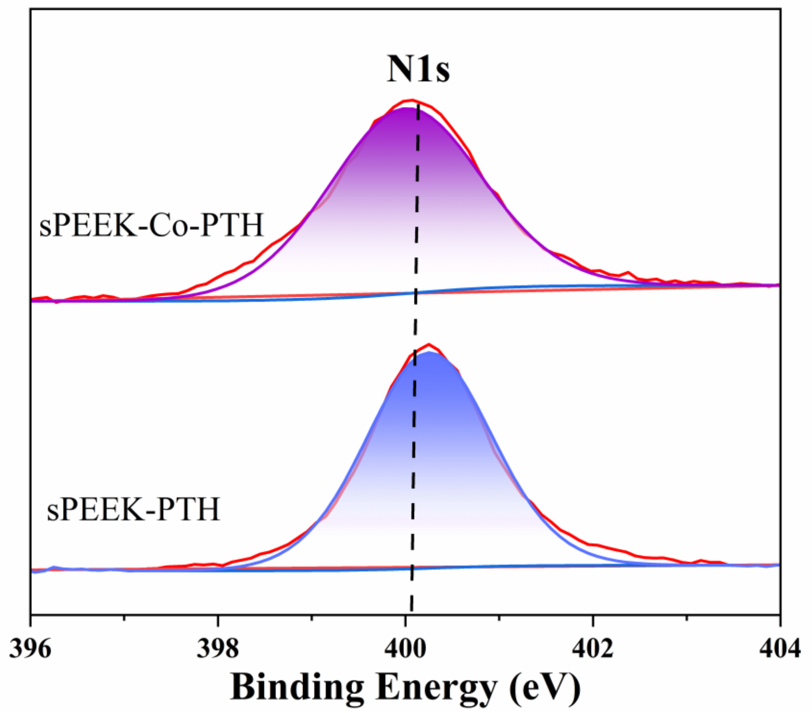

Figure S3: The high-resolution N 1s XPS spectra of the sPEEK-PTH and sPEEK-Co-PTH groups.

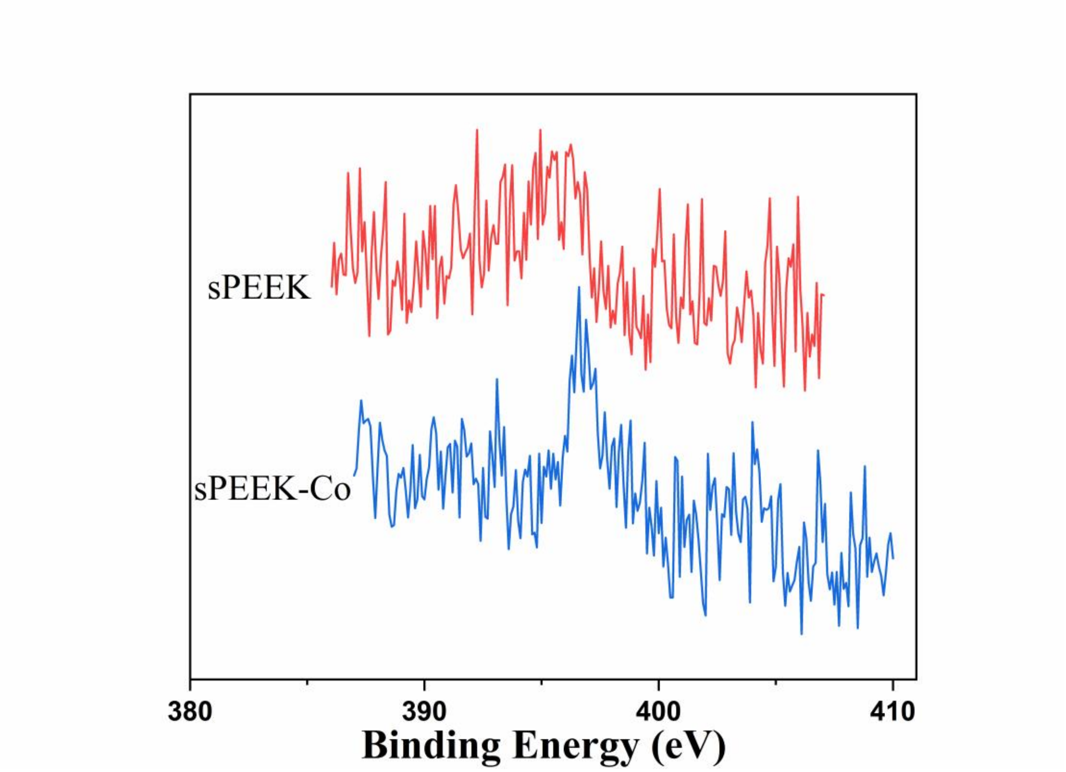

Figure S4: The high-resolution N 1s XPS spectra of the sPEEK and sPEEK-Co groups.

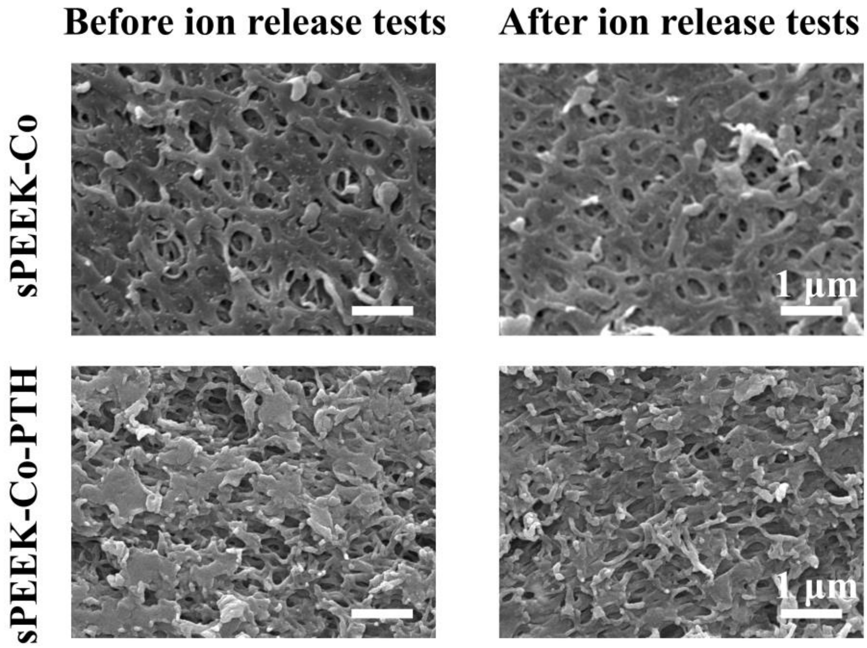

Figure S5: The surface morphology of sPEEK-Co and sPEEK-Co-PTH samples before and after ion release tests.

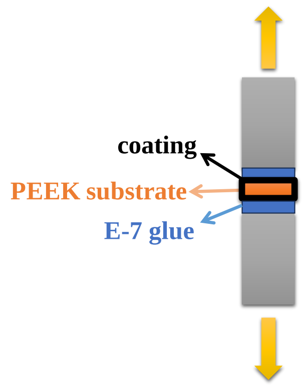

Figure S6: Schematic diagram of adhesion-tension test.

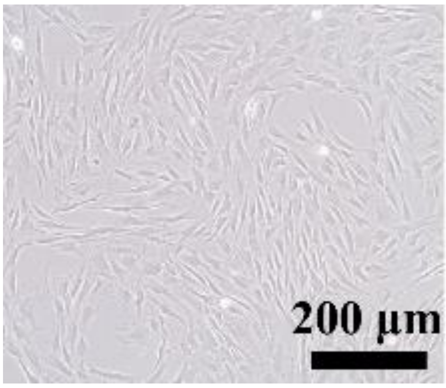

Figure S7: The microscopic image of the extracted BMSCs.

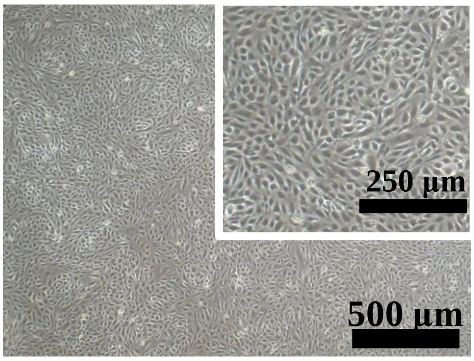

Figure S8: The microscopic images of human umbilical vein endothelial cells (HUVECs).

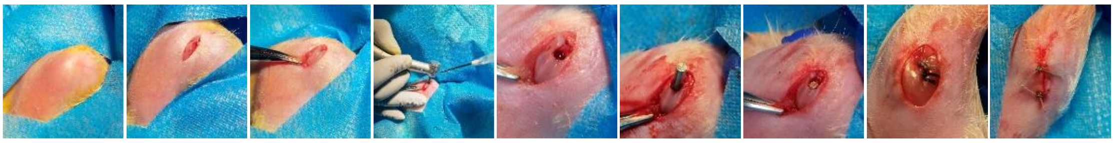

Figure S9: Intraoperative pictures of rat femur implantation experiment.

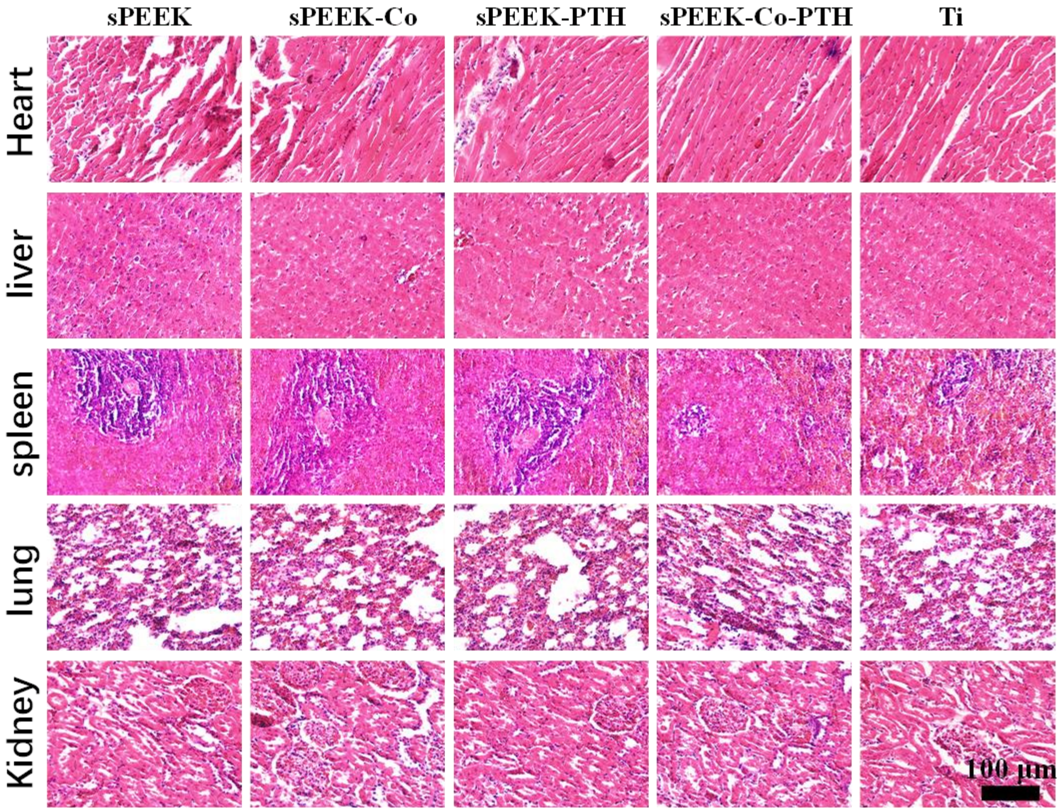

Figure S10: HE staining of heart, liver, spleen, lung, and kidney tissues after 4 weeks.

Table S1. Primer sequences of each gene in the RT-PCR analysis

Target gene	Forward primer (5’-3’)	Reverse primer (5’-3’)
ALP	ACAACACCAACGCTCAGGTC	GTGACCTCGTTCCCCTGAGT
RunX2	ACGAATGCACTATCCAGCCA	GCAGGTACGTGTGGTAGTGA
OPN	GGTGATAGCTTGGCTTACGGA	TGGCATCGGGATACTGTTCA
Col-1	CACTGCAAGAACAGCGTAGC	AAGTTCCGGTGTGACTCGTG
HIF-1α	GGCAGCAACGACACAGAAAC	TTTTCGTTGGGTGAGGGGAG
VEGF	GCAGAATCATCACGAAGTGGT	CCAGGGTCTCGATTGGATGG
bFGF	AAGGAGTGTGTGCTAACCGT	CTGCCCAGTTCGTTTCAGTG
SCF	GACCTTGTGGAGTGCGTGAA	CTGGGTTCTGGGCTCTTGAAT
